# Pulmonary syphilis with a cicatricial variant of organizing pneumonia: a case report

**DOI:** 10.1186/s12890-023-02469-6

**Published:** 2023-05-18

**Authors:** Ken Goda, Masahiro Katsurada, Takefumi Doi, Nobuyuki Saga, Yoshimasa Maniwa, Tsuneaki Kenzaka

**Affiliations:** 1 Department of Internal Medicine, Hyogo Prefectural Tamba Medical Center, 2002-7 Iso, Hikami- cho, Tamba, 669-3495 Japan; 2 Division of Community Medicine and Career Development, Kobe University Graduate School of Medicine, 2-1-5, Arata-cho, Hyogo-ku, Kobe, Hyogo 652-0032 Japan; 3 Department of Oncology Respiratory Medicine, Kita-harima Medical Center, 926-250 Ichiba-cho, Ono, 675-1392 Japan; 4 Department of Respiratory Medicine, Kobe University Graduate School of Medicine, 7-5-1 Kusunoki-cho, Chu-o-ku, Kobe, 650-0017 Japan; 5 Division of Thoracic Surgery, Kobe University Graduate School of Medicine, 7-5-2 Kusunoki-cho, Chuo-ku, Kobe, 650-0017 Japan; 6 Department of Diagnostic Pathology, Kobe University Hospital, 7-5-2 Kusunoki-cho, Chu-o-ku, Kobe, 650-0017 Japan

**Keywords:** *Treponema pallidum*, Syphilis, Lung, Cicatricial variant of organizing pneumonia

## Abstract

**Background:**

Syphilis is a chronic disease that progresses in the primary, secondary, latent, and tertiary stages. Pulmonary manifestations of syphilis are rare, and their histological features have not been well-described.

**Case presentation:**

A 78-year-old man was referred to our hospital because of a solitary nodular shadow in the right middle lung field on a chest radiograph. Five years prior, a rash appeared on both legs. He was tested for syphilis at a public health center, and the non-treponemal test result was negative. When he was approximately 35 years old, he had unspecified sexual intercourse. Chest computed tomography showed a 13-mm nodule with a cavity in S6 of the right lower lobe of the lung. Robot-assisted resection of the right lower lobe was performed because of suspected localized right lower lobe lung cancer. A cicatricial variant of organizing pneumonia (CiOP) was observed, and immunohistochemistry identified *Treponema pallidum* inside the macrophages in the nodule cavity. The rapid plasma regain (RPR) value was negative, and the *Treponema pallidum* hemagglutination assay was positive. The patient was diagnosed as having secondary syphilis with pulmonary involvement. Insidious progression of secondary syphilis may result in CiOP and a negative RPR test result.

**Conclusions:**

We report the first case of pulmonary syphilis with a histological pattern of CiOP. It may be asymptomatic and difficult to diagnose because the RPR test may be negative for a long period of time. When either non-treponemal or treponemal test results are positive, the possibility of pulmonary syphilis should be considered along with appropriate medical treatment.

## Background

Syphilis is a chronic disease caused by *Treponema pallidum;* the mode of transmission is usually sexual contact, but it may also be transmitted from mother to child and rarely through transfused blood [1]. The four stages of acquired syphilis are often known as primary, secondary, latent, and tertiary [2].

Clinical manifestations of secondary syphilis include rash and condyloma latum in intertriginous areas, lymphadenopathy, subclinical hepatitis, and systemic symptoms such as fever, malaise, and weight loss [2]. However, pulmonary manifestations of syphilis are rare, and their histological features have not been well-described [3].

A cicatricial variant of organizing pneumonia (CiOP) is a recently recognized subtype of organizing pneumonia, where the organizing granulation tissue within the alveolar space (“Masson polyp”) shows maturation to irreversible dense mature eosinophilic collagenous fibers [4]. Patterns on imaging that are not often linked with organizing pneumonia include linear abnormalities, reticular abnormalities, and dendriform pulmonary ossification. These instances demonstrate that CiOP should be included in the differential diagnosis for these imaging presentations [5]. Patients with CiOP seem to follow a passive and benign course on radiologic and clinical follow-up [6].

We report a case of secondary syphilis with pulmonary involvement and a histological pattern of CiOP. We aimed to clarify the association between syphilitic pulmonary nodules and CiOP.

## Case Presentation

A 78-year-old man visited our hospital because a chest radiograph taken during his medical check-up showed a solitary nodular shadow in the right middle lung field. Five years ago, a rash appeared on both legs, and he was tested for human immunodeficiency virus (HIV) and syphilis (non-treponemal test) at a public health center; the results were negative. One year prior, a chest radiograph at his medical check-up showed no abnormalities. Six months prior, he developed a mild cough; however, there was no fever, weight loss, or sputum.

He smoked 20 cigarettes per day from 19 to 28 years of age, drank alcohol (sake 270 mL/day), and had no history of asbestos exposure or tuberculosis. He was sexually active with an unspecified number of people when he was about 35 years old but was not currently sexually active. He had two children, and his wife had no abnormalities noted in the screening test for syphilis (non-treponemal test) at the time of their children’s birth.

### Investigations

His body temperature was 35.8 °C, blood pressure was 159/75 mmHg, pulse rate was 99 beats/min, and respiration rate was 12 breaths/min with a peripheral capillary oxygen saturation level of 98% (room air). No skin rashes or erythema was observed. There were no abnormal lung sounds, heart sounds, or cranial neurological findings.

Blood test results were as follows: white blood cell count was 6700 /µL with 74.3% neutrophils, C-reactive protein level was 0.05 mg/dL, carcinoembryonic antigen level was 5.88 ng/mL (standard value: ≤5.0 ng/mL, cytokeratin 19 fragment level was 1.3 ng/mL (≤ 3.5 ng/mL, progastrin-releasing peptide level was 66.0 pg/mL (< 46.0 pg/mL), and (1,3)-beta-d-glucan level was < 4.0 pg/mL (≤ 20.0 pg/mL). Aspergillus antigen and cryptococcal antigen test results were negative, interferon-gamma release assays were negative, rapid plasma reagin (RPR) value was under 0.4 RPR Units (R.U.) (< 1.0 R.U.), *Treponema pallidum* hemagglutination assay (TPHA) value was 60 Titer Units (T.U.) (< 10 T.U.), and HIV antigen and antibody test results were negative.

**Fig. 1:**
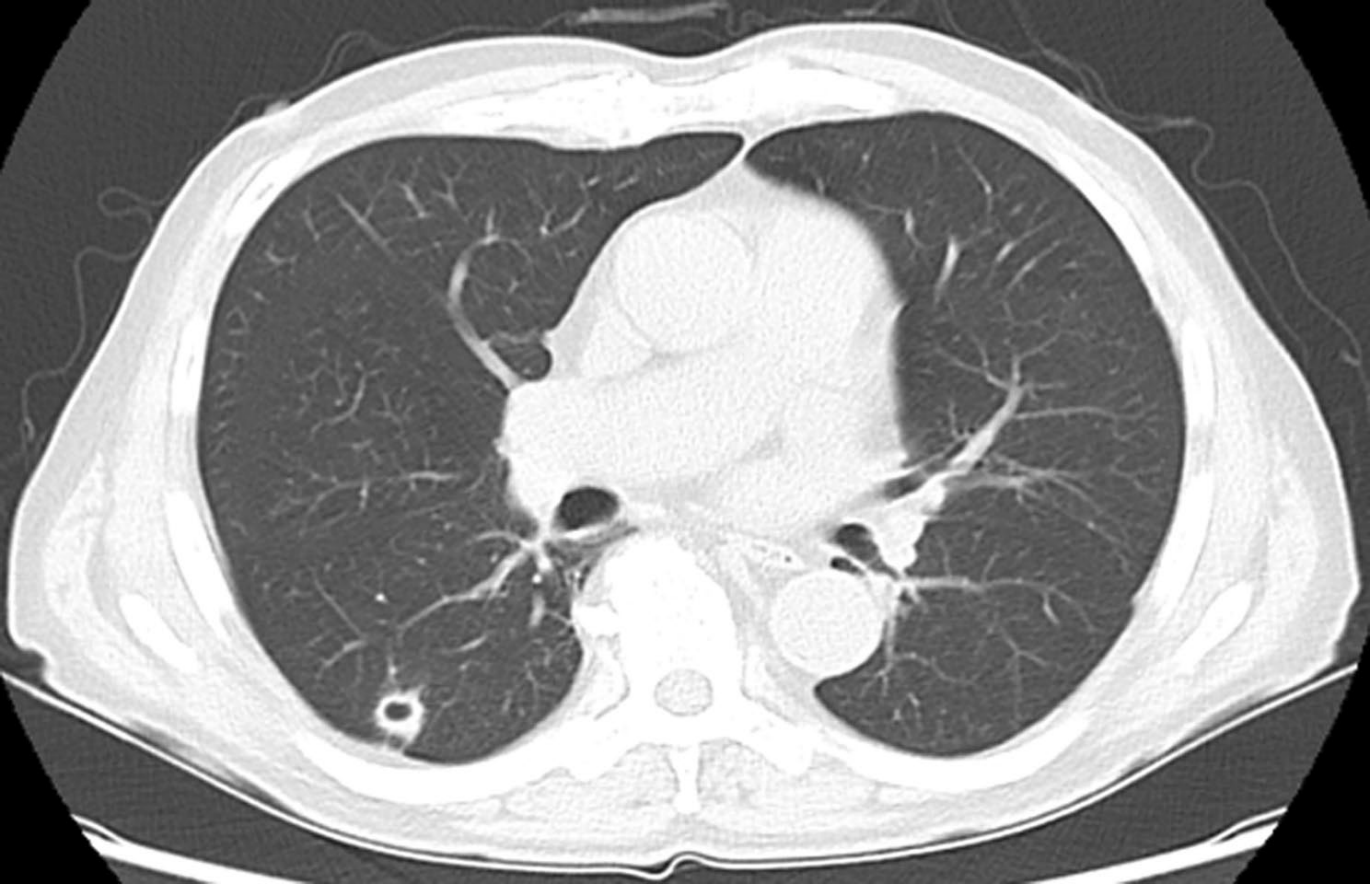
Chest computed tomography A 13-m nodule with a cavity is visible in S6 of the right lower lobe of the lung. The cavity wall was irregular and had a ground-glass opacity around it

**Fig. 2:**
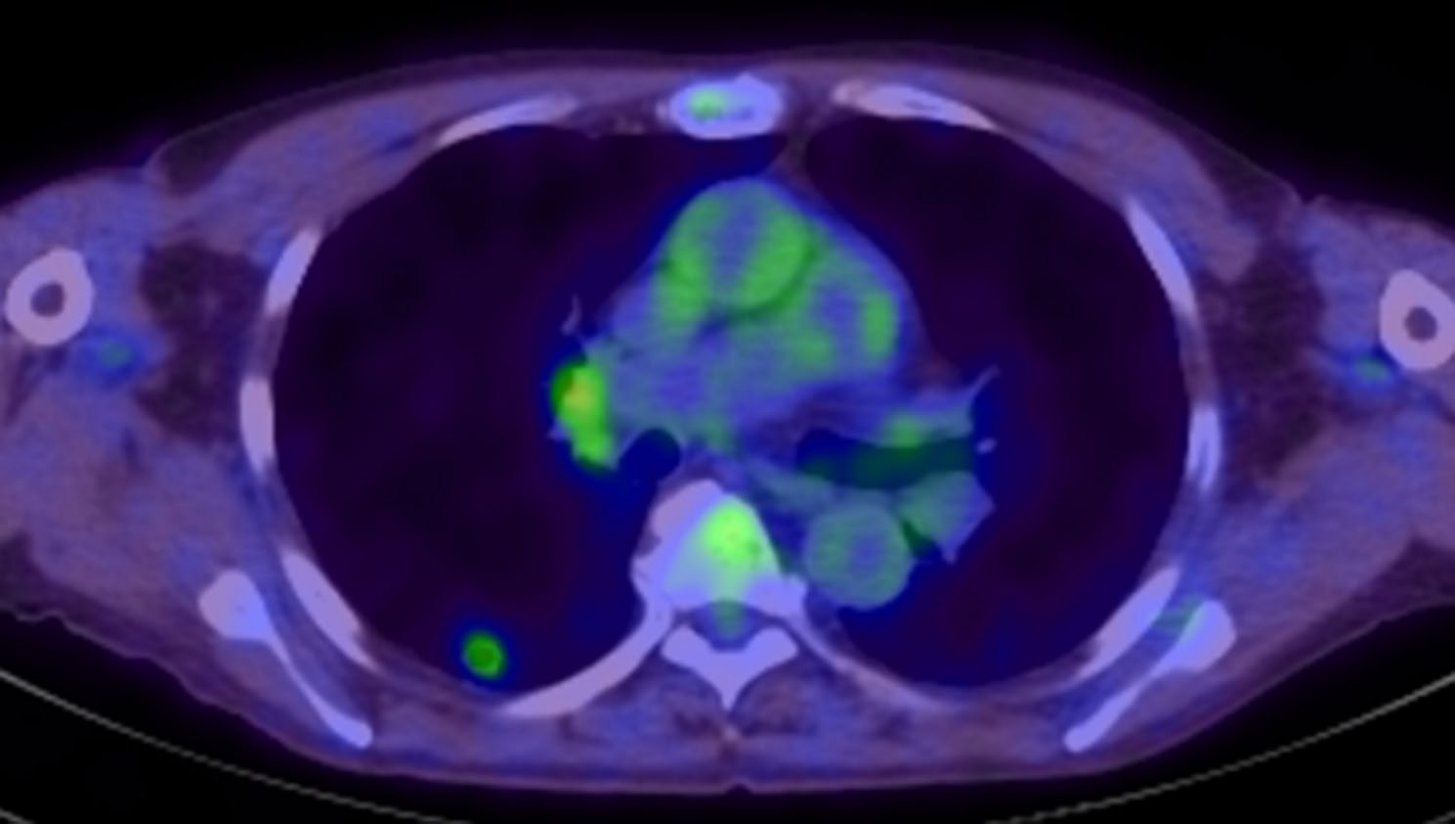
18 F-fluorodeoxyglucose positron emission tomography/computed tomography (FDG-PET/CT) FDG-PET/CT revealed a nodule with an internal cavity and 18 F-fluorodeoxyglucose accumulation (SUVmax = 2.25). The right hilar lymph node also showed 18 F-fluorodeoxyglucose accumulation (maximum standardized uptake value = 2.84)

Chest computed tomography (CT) showed a 13-mm nodule with a cavity in S6 of the right lower lobe of the lung (Fig. 1). The cavity wall was irregular and had a ground-glass opacity around it. 18 F-fluorodeoxyglucose positron emission tomography/computed tomography (FDG-PET/CT) revealed a nodule with an internal cavity and FDG accumulation (standardized uptake values (SUV) max = 2.25). The right hilar lymph node also showed FDG accumulation (SUVmax = 2.84) (Fig. 2).

### Differential diagnosis and treatment

**Fig. 3:**
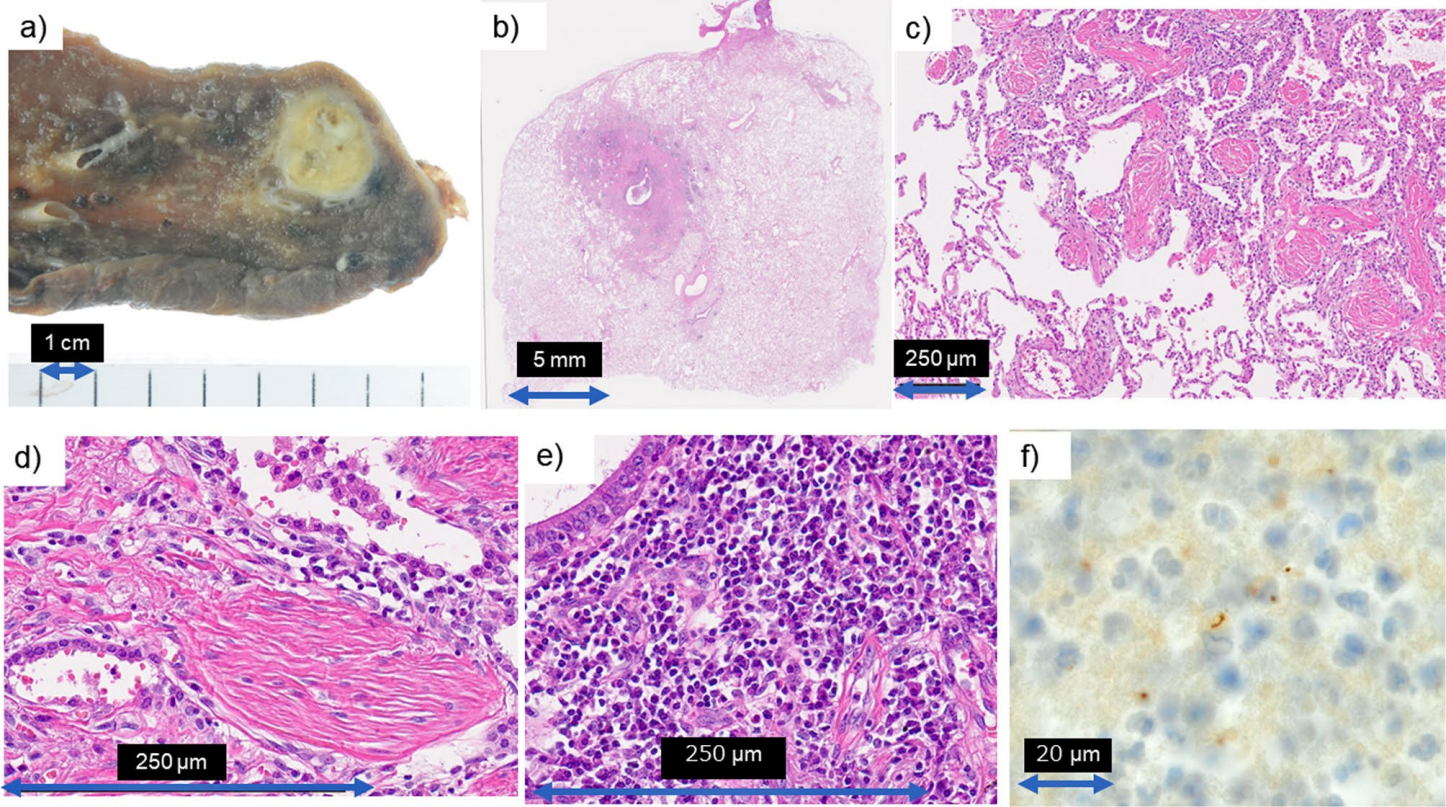
Pathological examination a): Right lower lobectomy was performed The cut surface revealed a yellow tan-colored nodule approximately 15 mm in size b): Nodules approximately 15 mm in size are seen with dilation of the bronchioles (original magnification ×1, hematoxylin and eosin staining) c): Around the margin of the nodule, many hyalinized fibrotic plugs were observed inside the alveolar space. They were interpreted as cicatricial variants of organizing pneumonia (original magnification ×40, hematoxylin and eosin staining) d): Higher magnification revealed plasmacytic infiltration around the dilatated bronchiole (original magnification ×400, hematoxylin and eosin staining) e): Deposition of collagenous fiber in Masson type polyp is a characteristic feature of cicatricial variant of organizing pneumonia (original magnification ×400, hematoxylin and eosin staining) f): Immunohistochemistry of *Treponema pallidum* revealed spiral fungi in the necrotic debris of dilated bronchioles

Lung cancer (clinical stage: cT1bN1M0) was suspected, and a robot-assisted right lower lobectomy with lymph node dissection was performed. Pathological examination revealed that the nodules were bronchiolocentric lesions, and inflammatory changes with lymphoplasmacytic infiltration and deposition of hyalinized collagen fibers with focal organizing pneumonia were noted. The cavity found on CT showed a dilated bronchiole with erosion. Immunohistochemistry (*T. pallidum*, polyclonal antibody, BioSource International, Inc, Hopkinton, MA, USA) revealed *T. pallidum* within necrotic debris in the cavity and inside macrophages around the inflammatory nodules. Cancer was not diagnosed, and no fungal or acid-fast bacillus infections were observed (Fig. 3). The lymph nodes showed reactive lymphadenopathy, and immunostaining was negative for syphilis. We diagnosed the patient with secondary syphilis with pulmonary involvement based on the positive serological TPHA levels and immunostaining results.

### Treatment and outcome

The patient received intramuscular benzathine benzylpenicillin (2.4 million Units), which was repeated 3 weeks later.

### Outcome and follow-up

There was no recurrence of the disease; the RPR value was 0.3 R.U. and the TPHA value was 41.9 T.U. 6 months after antibiotic treatment.

## Discussion and conclusions

We report a case of secondary syphilis with pulmonary involvement and a histological pattern of CiOP. To our knowledge, this is the first report of pulmonary syphilis with a histological pattern of CiOP.

Syphilis that affects the lungs is uncommon [3, 7] and can be congenital, secondary, or tertiary in nature. Coleman et al. proposed the following diagnostic criteria: physical and historical findings typical of syphilis, serological positivity for syphilis, pulmonary abnormalities on radiographs, exclusion of other forms of pulmonary disease, and radiologic response to antimicrobial therapy [7]. Diagnosing pulmonary involvement in secondary syphilis is challenging. Radiographic pulmonary lesions may appear as subpleural nodular opacities that are solitary or multiple and rarely infiltrate, pleural effusion, or lymphadenopathy [8–10]. The histological characteristics of secondary syphilis with pulmonary involvement have not been well-documented, despite a large number of cases. The confirmation of syphilis has been previously reported using serology, immunohistochemistry, or polymerase chain reaction for *T*. *pallidum*. Lung biopsy was frequently not performed or included, and treatment was determined based on serological confirmation and related clinical characteristics [3]. Recently, there have been reports of pulmonary syphilis diagnoses, and the use of genetic techniques to treat these cases [11, 12]. However, genetic techniques are not commonly used due to their high cost; immunological validation used in this case was inexpensive and easy to perform.

Similar to previous reports, the histological features of the current case were chronic inflammatory processes with lymphoplasmacytic infiltration and hyalinized collagenous fibrotic scars [3]. The characteristic pathological finding of the current case was CiOP in the marginal area of the nodules. Conventional organizing pneumonia is an edematous fibrous plug inside the alveolar space that is related to the acute/subacute inflammatory setting. However, in CiOP, the subsequent changes are a chronic process, with the deposition of hyalinized collagen fibers within the plug, eventually forming CiOP [4]. Lung lesions associated with syphilis range from asymptomatic to symptomatic, and imaging findings can be variable, for example, mass with multiple subpleural nodules and bilateral basilar reticulonodular infiltrates, multiple cavitating round nodules bilaterally. Although abscess formation has been observed in compromised host, who had received corticosteroid therapy for minimal lesion nephrotic syndrome [11], the history of CiOP in this case appears to be the result of chronic inflammation. As syphilis has a long-term course, the histological picture may have been different from the conventional OP observed in other rapid infectious diseases. To our knowledge, this is the first report of pulmonary syphilis with a histological pattern of CiOP. CiOP is a recently recognized subtype of organizing pneumonia; these histological changes may reflect the insidious progression of secondary syphilis.

In many cases, syphilis is strongly suspected, requiring appropriate initial testing. However, diagnosis of cases that have no clinical suspicion of syphilis is difficult [3]. *T. pallidum* culture is difficult and is usually used only in clinical or basic research [13]. Therefore, serum data are used to diagnose syphilis in clinical practice. The conventional serological testing algorithm for syphilis involves initial screening with a non-treponemal test (e.g., RPR). Non-treponemal reactivity testing is then confirmed with a treponemal test such as fluorescent treponemal antibody absorptiometry [14]. In this case, a discrepancy was observed, with a positive treponemal test and a negative non-treponemal test. False-positive treponemal tests can also be seen in a variety of other diseases, including spirochete infections, malaria, and leprosy [15]. In a report from New York, 3% of 116,822 specimens for first screening with a treponemal test and then retesting reactive results with a non-treponemal test were discordant [16]. Discordant results are often observed in patients with a history of successful syphilis treatment. For patients without a history of treated syphilis, a discordant result can occur in very early-stage syphilis or in cases with a prozone phenomenon [17]; in late-stage syphilis when non-treponemal tests have become nonreactive over time [18]; and in patients with advanced immunosuppression (e.g., patients with acquired immunodeficiency syndrome), which is thought to reflect B-cell failure during late-stage HIV infection [19, 20].

In the present case, based on the patient’s history and physical and laboratory findings, he was not infected with HIV, and there was no skin rash, oral lesions, or lymphadenopathy characteristic of secondary syphilis. Although secondary syphilis was possible due to the skin rash 5 years prior, a negative RPR test at the health center was reported. Thus, the patient was unable to seek medical attention and undergo the treponemal test. It is considered that the patient was infected at the age of 35 years, followed by a period of latency. Five years ago, when he was 73 years of age, he had recurrent secondary syphilis, presenting as skin lesions. The patient had recurrent secondary syphilis (skin and lung lesions) and latent syphilis; however, we could not detect and diagnose syphilis using RPR screening because of the long period of time. A variety of radiographs and CT images can be taken for pulmonary syphilis. When either non-treponemal or treponemal test results are positive, the possibility of pulmonary syphilis should be considered along with appropriate medical treatment.

In summary, we report the first case of pulmonary syphilis with a histological pattern of CiOP. It may be asymptomatic and difficult to diagnose because the RPR test may be negative for a long period of time. When either non-treponemal or treponemal test results are positive, the possibility of pulmonary syphilis should be considered along with appropriate medical treatment.

## Data Availability

All data supporting our findings are contained within this published article.

## Abbreviations

CiOP: Cicatricial variant of organizing pneumonia
RPR: Rapid plasma reagin
R.U.: RPR Units
TPHA: *Treponema pallidum* hemagglutination assay
T.U.: Titer Units
CT: Computed tomography
FDG-PET/CT: 18 F-fluorodeoxyglucose positron emission tomography/computed tomography
HIV: human immunodeficiency virus
SUV: standardized uptake values
